# COVID-19 Vaccine-Induced Parsonage-Turner Syndrome: A Case Report and Literature Review

**DOI:** 10.7759/cureus.25493

**Published:** 2022-05-30

**Authors:** Mohammad Asim Amjad, Zamara Hamid, Yamini Patel, Mujtaba Husain, Ammad Saddique, Adnan Liaqat, Pius Ochieng

**Affiliations:** 1 Internal Medicine, The Wright Center for Graduate Medical Education, Scranton, USA; 2 Medicine, Shifa International Hospital, Islamabad, PAK; 3 Internal Medicine, Southeast Health Medical Center, Dothan, USA; 4 Pulmonary and Critical Care Medicine, Geisinger Community Medical Center, Scranton, USA

**Keywords:** post covid vaccination parsonage-turner syndrome, covid 19, neuralgic amyotrophy, sars-cov-2 vaccines, acquired peripheral neuropathy

## Abstract

All modern vaccines share the risk of neurological adverse effects. Only a few cases of Parsonage-Turner syndrome (PTS), an uncommon peripheral nerve condition associated with coronavirus disease 2019 (COVID-19) immunization, have been reported to date. We describe a case of COVID-19 vaccine-induced PTS and provide a brief literature review. A 78-year-old male non-smoker with a medical history of coronary artery disease presented with non-exertional, constant chest pain for one hour and new onset of bilateral hand weakness for three days. He had no neurological disease or allergies and denied any recent trauma or infection. Three weeks before the onset of the symptoms, the patient received a second dose of the BNT162b2 COVID-19 vaccine, which was administered 21 days after the first dose. Physical examination was significant for weakness in right-hand grip and wrist flexion. There were no other motor deficits, upper motor neuron signs, bulbar weakness, or sensory deficits. Diagnostic workup for the underlying diabetes mellitus, infections, or other autoimmune diseases was negative. Imaging workup revealed no demyelination, fracture deformity, traumatic subluxation, or compressive myelopathy. Nerve conduction studies, including needle electromyography, showed decreased motor unit recruitment in the bilateral first dorsal interosseous and right deltoid, biceps, and triceps muscles confirming PTS. The patient was treated with 40 mg/day of oral prednisone and occupational therapy to maintain range of motion and activities of daily living. PTS is also known as neuralgic amyotrophy, brachial plexus neuritis, brachial plexopathy, and shoulder-girdle syndrome. It is characterized by asymmetrical, chronic, resistant upper extremity neuropathic pain and neurological defects such as paralysis and paresthesia. There are two different types of PTS: non-hereditary and inherited. The etiology and pathophysiology of PTS are not fully understood. Various aspects such as genetic, environmental, and immunological predisposition may play a role in developing the syndrome. Infections, vaccines, and injuries are typical causes of non-hereditary forms. After the COVID-19 epidemic and the commencement of a global immunization effort, similar instances happened. Presently there is no available test that unequivocally confirms or excludes PTS itself. Electrodiagnostic study and imaging modalities help to rule out other differential diagnoses. Also, there is no specific treatment available; however, it may resolve independently of treatment with supportive care.

## Introduction

Parsonage-Turner syndrome (PTS), also called neuralgic amyotrophy, was first described in 1948 by Parsonage et al. in a series of case studies [1]. A condition has bouts of neuropathic pain, fast multifocal paresis, and atrophy of the upper extremities, with a long prognosis and recovery period of years [2]. Following vaccination, the first-ever case was reported in 1956 by Rigal et al. [3]. We present a similar unique case of PTS following COVID-19 vaccination and add a brief literature review to the documentation of this rare disease.

## Case presentation

**Figure 1:**
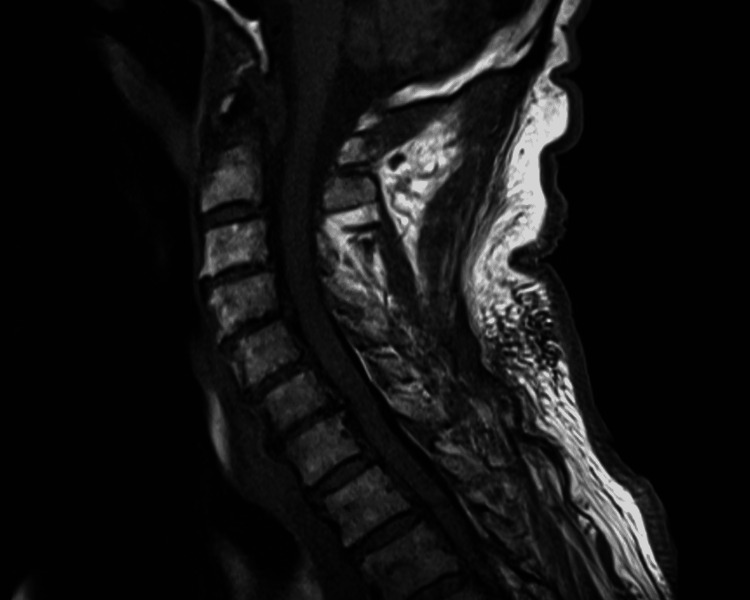
MRI of the cervical spine, showing multilevel degenerative disc disease and no signs of demyelination, fracture deformity, traumatic subluxation, or compressive myelopathy

A 78-year-old male non-smoker with a medical history of coronary artery disease presented with non-exertional, constant chest pain for one hour and new onset of bilateral hand weakness for three days. He had no neurological disease or allergies and denied any recent trauma or infection. Three weeks before the onset of the symptoms, the patient received a second dose of the BNT162b2 COVID-19 vaccine, which was administered 21 days after the first dose. Physical examination was significant for weakness in right-hand grip and wrist flexion (Medical Research Council grade 3). There were no other motor deficits, upper motor neuron signs, bulbar weakness, or sensory deficits. Diagnostic workup for the underlying acute coronary syndrome, infection (cytomegalovirus, Epstein-Barr virus, HIV, mycoplasma, Lyme disease), and rheumatological diseases (antinuclear antibody, rheumatoid factor, Sjögren syndrome A (Ro) or Sjögren syndrome B (La), antineutrophil cytoplasmic antibody) were negative. Imaging workup including magnetic resonance imaging (MRI) brain, cervical spine, and thoracic spine done with and without contrast revealed no demyelination, fracture deformity, traumatic subluxation, or compressive myelopathy (Figure 1). Nerve conduction studies showed brachial plexopathy on the left side of the lower trunk, bilateral median neuropathies at the wrist, and ulnar sensory neuropathy.

Needle electromyography showed decreased motor unit recruitment in the bilateral first dorsal interosseous and right deltoid, biceps, and triceps muscles (Tables 1-3). The patient was treated with 40 mg/day of oral prednisone and occupational therapy to maintain range of motion and activities of daily living. He showed significant improvement in pain, with a slight recovery from his weakness. He was then discharged with regular outpatient follow-ups to observe the disease course and taper steroids.

**Table 1:** Sensory nerve conduction study findings Bilateral median sensory nerve action potential (SNAP) amplitudes are diminished, and peak latency is prolonged on the left and normal on the right. Bilateral ulnar sensory nerve action potential (SNAP) amplitudes are diminished, severely on the right; peak latencies are borderline. Bilateral radial sensory nerve action potential (SNAP) amplitudes are borderline, and peak latencies and conduction velocities are normal. Bilateral ulnar compound muscle action potential (CMAP) amplitudes and distal latencies are normal. L - left; R - right

Nerve/sites	Recruitment site	Onset latency ms	Peak latency ms	Reference ms	NP amplitude µV	Reference µV	Segments	Distance mm	Velocity m/s	Reference m/s
L median - Digit II (antidromic)										
Wrist	Digit II	3.5	4.4	≥3.6	7.4	≥15.0	Wrist - Digit II	130	37	≥56
R median - Digit II (antidromic)										
Wrist	Digit II	2.8	3.5	≤3.6	7.5	≥15.0	Wrist - Digit II	130	46	≥56
L ulnar - Digit V (antidromic)										
Wrist	Digit V	2.6	3.2	≤3.1	6.2	≥10.0	Wrist - Digit V	110	49	≥54
R ulnar - Digit V (antidromic)										
Wrist	Digit V	2.6	3.2	≤3.1	1.9	≥10.0	Wrist - Digit V	110	43	≥54
L radial - anatomical snuffbox (forearm)										
Forearm	Wrist	1.8	2.5	≤2.9	20.9	≥20.0	Forearm - wrist	100	56	≥49
R radial - anatomical snuffbox (Forearm)										
Forearm	Wrist	1.5	2.2	≤2.9	21.2	≥20.0	Forearm - wrist	100	69	≥49
L median, ulnar - transcarpal comparison										
Median palm	Wrist	2.3	3.1	≤2.3	24.6	≥50	Median palm - wrist	80	35	≥56
Ulnar palm	Wrist	1.4	2.0	≤2.3	8.8	≥15.0	Ulnar palm - wrist	80	59	≥56
R median, ulnar - transcarpal comparison										
Median palm	Wrist	2.1	2.8	≤2.3	21.9	≥50.0	Median palm - wrist	80	38	≥56
Ulnar palm	Wrist	1.4	2.2	≤2.3	3.0	≥15.0	Ulnar palm - wrist	80	59	≥56

**Table 2:** Motor nerve conduction study revealed no significant changes L - left; R - right; APB - abductor pollicis brevis; ADM - abductor digiti minimi; FDI - first dorsal interosseous (muscle); A - above; B - below

Nerve/Sites	Latency ms	Reference ms	Amplitude mV	Reference mV	Relative amplitude %	Duration ms	Segments	Distance mm	Latency difference ms	Velocity m/s	Reference m/s
L median - APB											
Wrist	4.5	≤4.5	3.0	≥4.0	100	5.0	Wrist - APB	70			
Elbow	9.6		2.8		93.2	5.1	Elbow - wrist	240	5.1	47	≥48
Ulnar wrist	3.2		2.1	76.4	6.4						
Ulnar elbow	9.4		1.8	84	7.2						
R median - APB											
Wrist	4.0	≤4.5	5.0	≥4.0	100	6.8	Wrist - APB	70			
Elbow	8.5		4.9		98.1	7.2	Elbow - wrist	235	4.5	52	≥48
L ulnar - ADM											
Wrist	2.8	≤3.6	11.9	≥6.0	100	6.4	Wrist - ADM	65			
B elbow	7.0		9.3		93.6	6.7	B elbow - wrist	230	4.5	55	≥51
A elbow	9.0		9.0		96	6.1	A elbow - wrist	330	6.2	53	≥51
R ulnar - ADM											
Wrist	2.6	≤3.6	11.9	≥6.0	100	6.4	Wrist - ADM	65			
B elbow	7.1		11.2		93.9	6.7	B elbow wrist	230	4.5	51	≥51
A elbow	9.3		11.2		99.9	6.9	A elbow	330	6.8	49	≥51
L ulnar - FDI											
Wrist	3.5	≤3.7	11.5	≥7.0	100	5.2	Wrist FDI				
B elbow	7.8		10.7		93.4	5.7	B elbow wrist	230	4.2	55	≥51
A elbow	9.7		10.7		99.3	5.8	A elbow	330	6.2	53	
R ulnar - FDI											
Wrist	3.3	≤3.7	13.3	≥7.0	100	5.2	Wrist FDI				
B elbow	7.8		12.2		91.9	5.6	B elbow wrist	230	4.5	51	≥51
A elbow	9.9		11.5		94.6	5.7	A elbow	330	6.7	50	

**Table 3:** Electromyography summary table Electromyography (EMG) reveals decreased motor unit recruitment in the affected region's hallmark for Parsonage-Turner syndrome (bilateral first dorsal interosseous and right deltoid, biceps, and triceps muscles). L - left; R - right; MUAP - motor unit action potential; PSW - positive sharp waves

	Insertional	Spontaneous			MUAP					Additional
Muscle	Activity	Fibrillation​​​​​​/PSW	Fasciculations	Other	Duration	Amplitude	Polyphasia	Recruitment	Activations	Comments
L first dorsal interosseous	Normal	None	None	None	N	1	None	Reduced	Normal	Mild
R deltoid	Normal	1	Few	None	1	2	3	Reduced	Normal	Moderate
R biceps brachii	Normal	None	None	None	2	2	3	Reduced	Normal	Moderate to severe
R triceps brachii	Normal	None	None	None	N	1	None	Reduced	Normal	Mild
R flexor carpi ulnaris	Normal	None	None	None	N	Normal	1	Reduced	Normal	Mild
R first dorsal interosseous	Normal	None	None	None	N	-1	2	Single unit	Normal	

## Discussion

PTS is also known as neuralgic amyotrophy, brachial plexus neuritis, brachial plexopathy, and shoulder-girdle syndrome. Parsonage et al. were the pioneers to describe 136 cases of brachial plexus neuropathy or its synonyms in 1948 [1]. Over the years, a number of publications have been published on the clinical spectrum of PTS. Van Alfen provided a very detailed case series involving 246 individuals in a healthcare setting [3]. They defined the clinical characteristics of this disease process, which included severe, relentless, asymmetrical upper extremity neuropathic pain, which may progress to neurological deficits such as weakness and paresthesia, often in the distribution of individual nerves. 

PTS can be classified into non-hereditary and hereditary forms. The latter form is due to mutation of the SEPT9 gene on chromosome 17q25 in about 85% of cases [4]. The overall incidence rate is more common in men (68%), estimated at two to four cases per 100,000 population per year [5]. Rigal et al. were the first to report PTS following vaccination [2]. Most occurred after polio, chickenpox, hepatitis B, influenza, and HPV immunizations. However, post-vaccination PTS is an infrequent entity, with only 4.3-15% of all cases being attributed to vaccines [5]. Similar cases have been reported with the advent of the COVID-19 pandemic and the initiation of a robust global vaccination drive. 

Generally, PTS is a form of peripheral neuropathy that encompasses any disorder that exclusively affects the nerves outside the central nervous system. PTS embodies a wide-ranging spectrum of clinical expressions. Classically, the patient presents with severe upper extremity pain, followed by neurological deficits. The extent and distribution of the affected peripheral nerve's pathology vary, including lumbosacral plexus, phrenic nerve, and recurrent laryngeal nerve during the attack [3,5,6]. 

The etiology and pathophysiology of PTS are not fully understood. Various aspects such as genetic, environmental, and immunological predisposition may play a role in developing the syndrome. Most cases arise from an autoimmune response (infectious or environmental triggers) that initiates inflammation of selected peripheral nerves by lymphocytic infiltrates, causing axonal degeneration and leading to their constriction [7]. 

Table 4 indicates the most common triggering factors described in the previous studies. Some investigators postulated that several affected persons are genetically susceptible to developing PTS following such exposures as described. An individual with a genetic predisposition may not express the disease unless and until they are exposed to environmental or immunological conditions that trigger its activation [3,8].

**Table 4:** Common causes of Parsonage-Turner syndrome PAN - polyarteritis nodosa References: [3,8]

Common causes of Parsonage-Turner syndrome
1. Idiopathic
2. Hereditary
3. Infection (viral, bacterial, parasitic)
4. Brachial plexus surgery
5. Unaccustomed strenuous exercise
6. Minor trauma
7. Anesthesia
8. Rheumatological diseases
9. Vaccinations (influenza, human papillomavirus, tetanus, hepatitis B, typhoid)
10. Autoimmune disorders (PAN, lupus, temporal arteritis)

Vaccines elicit potent systemic immune responses associated with an autoimmune reaction, possibly inducing PTS. Post-vaccination neuralgic amyotrophy develops within four weeks of its administration. Patients can present with symptoms in the contralateral to the injected region, indicating that PTS is unlikely to be attributable to direct nerve injury from the immunization [8]. Our literature review yielded seven reported cases following COVID-19 vaccination, which we have summarized along with our case here. 

Currently, the Food and Drug Administration has approved three COVID-19 vaccines; Moderna (mRNA-1273), Pfizer/BioNTech (BNT162b2), and Johnson & Johnson/Janssen (AD26.COV2.S). More than seven billion people worldwide have received at least one dose of the COVID-19 vaccine, including 417.80 million in the United States [9]. For neuralgic amyotrophy and brachial neuritis, Vaccine Adverse Event Reporting System (VERS) yielded 60 reports (29 for mRNA-1273, 30 for BNT162b2, and one for AD26.COV2.S) [10]. Significant limitations to this system are reporting bias, as it is open to the general population, and underreporting of the cases. 

There appeared to be gender predilection in our review of cases, with most patients being males. The mean age at presentation was 52, with the youngest patient being diagnosed at 35 years (Table 5). In all these cases, the most common presentation was pain, but the location of pain showed variability. Our case was unique, as he presented with atypical chest pain. Depending on the peripheral nerves affected, associated sensory symptoms in the form of paresthesia or numbness were present in most cases.

**Table 5:** Summary of previously published data

Author	Age	Sex	Time of onset	Vaccine	Treatment	Outcome
Queler et al. [11]	49	Male	13 hours	BNT162b2	Steroids	Improvement
	44	Male	18 hours	mRNA-1273	Gabapentin	Improvement
Mahajan et al. [12]	50	Male	One week	BNT162b2	Steroids	Improvement
Crespo Burillo et al. [13]	38	Male	Four days	Astra Zeneca	Steroids	Improvement
Diaz-Segarra et al. [14]	35	Female	Nine days	BNT162b2	Steroids	Improvement
Waheed et al. [15]	57	Female	One week	BNT162b2	Gabapentin	Improvement
Coffman et al. [16]	66	Female	Four weeks	BNT162b2	Steroids	Improvement
Our case	78	Male	Four weeks	BNT162b2	Steroids	Improvement

Presently there is no available test that unequivocally confirms or excludes PTS itself. Electrodiagnostic study and imaging modalities (MRI, ultrasound) help to rule out other differential diagnoses [17]. As PTS is regarded to be an axonal injury, needle electromyography can assist in determining axonal damage and reinnervation. Electromyography needs to be detail-oriented on the specific muscles of the upper limb. In all these studies, including in our case report, there was decreased motor unit recruitment in the affected muscles (Table 3). Also, MR neurography and high-resolution ultrasound are valuable diagnostic tools [7,18]. Two of the cases by Queler et al. depicted their significance in early detection [11]. 

There is no specific treatment for PTS, as it may resolve independently of treatment [17]. During acute phases, supportive pain management strategies such as non-steroidal anti-inflammatory drugs and opiates are beneficial, while most patients are managed conservatively. Some researchers recommend the administration of oral prednisone early in the course of the disease, which can help shorten the disease progression and assist in early recovery [19]. After the acute neuropathic pain, some physicians recommend using co-analgesics (amitriptyline, carbamazepine, gabapentin) instead of steroids to prevent steroid-related adverse effects [17,20]. Of eight reviewed cases, including ours, six received oral prednisone, while the remaining two were given gabapentin. All of them had significant improvement in symptoms. In addition, physical rehabilitation therapy also helped to cope with muscle weakness by preserving muscle strength and range of joint motion. Surgery (nerve decompression, reconstruction) can be considered in selected refractory cases and is beneficial if done within 6-12 months of injury [17]. 

COVID-19 vaccination drive is vital to ensure public health immunity against the SARS-CoV-2 viral infection. With increasing vaccination around the globe, more such cases will manifest. Awareness of this association is critical for timely recognition and management to improve clinical outcomes.

## Conclusions

Parsonage-Turner syndrome is a rare condition that presents with neuropathic pain and neurological deficits. More frequently observed after a mechanical injury, infection, or vaccination. The clinical examination, along with further evidence from electrodiagnostic (EMG) testing and imaging (MRI, high-resolution ultrasonography), is the primary method of diagnosis. With a comprehensive COVID-19 immunization campaign, physicians should be able to intervene promptly as the number of PTS patients rises. This typically resolves itself with a favorable prognosis.

## References

[1] Parsonage MJ, Turner JWA (1948). Neuralgic amyotrophy the shoulder-girdle syndrome. Lancet.

[2] Rigal Rigal, Bannel Bannel, Florentin Florentin, Parrot Parrot, Dinand Dinand (1956). Parsonage and Turner's neuralgic amyotrophy or shoulder syndrome; a new postvaccinal case (In French). J Med Bord.

[3] van Alfen N, van Engelen BG (2006). The clinical spectrum of neuralgic amyotrophy in 246 cases. Brain.

[4] Kuhlenbäumer G, Hannibal MC, Nelis E (2005). Mutations in SEPT9 cause hereditary neuralgic amyotrophy. Nat Genet.

[5] van Alfen N, van Eijk JJ, Ennik T (2015). Incidence of neuralgic amyotrophy (Parsonage Turner syndrome) in a primary care setting--a prospective cohort study. PLoS One.

[6] van Eijk JJ, Madden RG, van der Eijk AA (2014). Neuralgic amyotrophy and hepatitis E virus infection. Neurology.

[7] ArÁnyi Z, Csillik A, DéVay K, Rosero M, Barsi P, BÖhm J, Schelle T (2017). Ultrasonography in neuralgic amyotrophy: Sensitivity, spectrum of findings, and clinical correlations. Muscle Nerve.

[8] van Alfen N (2011). Clinical and pathophysiological concepts of neuralgic amyotrophy. Nat Rev Neurol.

[9] Ritchie H, Mathieu E, Rodés-Guirao L (2021). Coronavirus pandemic (COVID-19). https://ourworldindata.org/coronavirus.

[10] Chen RT, Rastogi SC, Mullen JR, Hayes SW, Cochi SL, Donlon JA, Wassilak SG (1994). The vaccine adverse event reporting system (VAERS). Vaccine.

[11] Queler SC, Towbin AJ, Milani C, Whang J, Sneag DB (2022). Parsonage-Turner Syndrome following COVID-19 vaccination: MR neurography. Radiology.

[12] Mahajan S, Zhang F, Mahajan A, Zimnowodzki S (2021). Parsonage Turner syndrome after COVID-19 vaccination. Muscle Nerve.

[13] Crespo Burillo JA, Loriente Martínez C, García Arguedas C, Mora Pueyo FJ (2021). Amyotrophic neuralgia secondary to Vaxzevri (AstraZeneca) COVID-19 vaccine. Neurologia.

[14] Diaz-Segarra N, Edmond A, Gilbert C, Mckay O, Kloepping C, Yonclas P (2021). Painless idiopathic neuralgic amyotrophy after COVID-19 vaccination: a case report. PM R.

[15] Waheed W, Carey ME, Tandan SR, Tandan R (2021). Post COVID-19 vaccine small fiber neuropathy. Muscle Nerve.

[16] Coffman JR, Randolph AC, Somerson JS (2021). Parsonage-Turner syndrome after SARS-CoV-2 BNT162b2 vaccine: a case report. JBJS Case Connect.

[17] Gstoettner C, Mayer JA, Rassam S (2020). Neuralgic amyotrophy: a paradigm shift in diagnosis and treatment. J Neurol Neurosurg Psychiatry.

[18] Sneag DB, Rancy SK, Wolfe SW, Lee SC, Kalia V, Lee SK, Feinberg JH (2018). Brachial plexitis or neuritis? MRI features of lesion distribution in Parsonage-Turner syndrome. Muscle Nerve.

[19] van Alfen N, van Engelen BG, Hughes RA (2009). Treatment for idiopathic and hereditary neuralgic amyotrophy (brachial neuritis). Cochrane Database Syst Rev.

[20] Feinberg JH, Doward DA, Gonsalves A (2007). Cervical radiculopathy vs Parsonage-Turner syndrome: a case report. HSS J.

